# Androgen receptor CAG repeats, non-random X chromosome inactivation, and loss of heterozygosity at Xq25 in relation to breast cancer risk

**DOI:** 10.1186/1471-2407-14-144

**Published:** 2014-03-01

**Authors:** Hui-Tzu Chen, Yao-Chung Wu, Shou-Tung Chen, Hsien-Chang Tsai, Yi-Chih Chien

**Affiliations:** 1 Comprehensive Breast Cancer Center, Changhua Christian Hospital, Changhua, Taiwan; 2 Department of Biology, National Changhua University of Education, No.1, Jin-De Road, 50058 Changhua City, Taiwan

**Keywords:** Breast cancer, Androgen receptor, Non-random X-chromosome inactivation, Loss of heterozygosity, Xq25

## Abstract

**Background:**

The aim of this study was to examine the association of non-random X chromosome inactivation (XCI) and loss of heterozygosity (LOH) at Xq25 with breast cancer development.

**Methods:**

Seventy-nine breast cancer patients, 39 female lung cancer patients, 30 other cancer patients and 77 healthy females were analysed for LOH using a panel of 11 microsatellite markers spanning Xq25. The androgen receptor (AR) gene was chosen as an XCI marker.

**Results:**

LOH of at least one microsatellite locus at Xq25 was identified in 46/65 breast cancers examined, while only 10/25 cancers of other origins demonstrated LOH in this region (*p* = 0.014). The critical deletion region in breast cancer was around marker DXS1047 (47.23%). Moreover, we found that tissues from eight breast cancers showed LOH at all of the informative loci tested at Xq25, while the other 38 showed partial (interstitial or telomeric) alterations at Xq25. Interestingly, the pattern of XCI of these eight breast cancers tended to be non-random. We estimated the frequencies of *AR* alleles and found that women with two long *AR* alleles (≥21 CAG repeats) had an increased risk of developing breast cancer, while those with two short *AR* alleles (<21 CAG repeats) were likely to be normal (*p* = 0.00069).

**Conclusions:**

The extraordinary high frequencies of LOH at Xq25 found in this study strongly imply that there might be one or more tumour suppressor genes (TSGs) related to the development of breast cancer at Xq25 in the Taiwanese female population.

## Background

Human female cells possess two X chromosomes, while male cells have only one. To compensate for X chromosome gene dosage, one X chromosome in each female cell condenses to form a Barr body. Genes on this chromosome are thus shut down in a phenomenon known as X chromosome inactivation (XCI). Which X chromosome undergoes condensation is generally random, therefore theoretically the ratio of paternal inactivation vs. maternal should be 1:1 in a given mass of cells. This phenomenon is known as Lyonization [1,2].

However, the pattern of XCI in some women is not random (non-random, or skewed Lyonization). Some research suggests that women with non-random XCI are more likely to be affected by X-linked recessive diseases, such as Wiskott-Aldrich syndrome [3], Hunter’s syndrome [4], Haemophilia [5], Duchenne’s muscular dystrophy [6], and Borjeson-Forssman-Lehmann syndrome [7]. Females are carriers of X chromosome mutations and do not usually express the phenotype because the normal allele can compensate for the defect, hence men are more likely to be affected by X-linked recessive diseases. However, non-random XCI in women can mimic the effects of the single X chromosome in men if the mutated X chromosome is active and the normal X chromosome is inactive, leading to expression of X-linked recessive diseases in women.

The androgen receptor (*AR*) gene has been used to demonstrate the pattern of X chromosome inactivation [8-10]. The 5′ CpG island of the *AR* gene is methylated on the inactive X chromosome and hypomethylated on the active X chromosome. Additionally, the *AR* alleles of paternal and maternal origins may be distinguished by a highly polymorphic CAG triplet repeat within the region of the CpG island. Moreover, the methylation status of a restriction site (GCGC) that is less than 100 bp away from the polymorphic CAG triplet repeat can be recognized by the methylation-sensitive restriction enzymes *HpaII* and *HhaI*[11]. Amplification of the CpG island region by polymerase chain reaction (PCR) following methylation-sensitive restriction enzyme digestion is possible only when the *AR* gene is fully methylated. Therefore, comparing the *AR* allele ratio with or without digestion of enzyme is a convenient method to determine whether the pattern of XCI is random or non-random.

The *AR* gene contains a polymorphic CAG trinucleotide repeat that encodes an uninterrupted polyglutamine tract in the N-terminal domain, ranging from 6–39 repeats in healthy individuals [12]. The number of CAG repeats has been linked with hormone-related cancers in both men and women. Several studies have demonstrated that shorter CAG repeat length is associated with increased prostate cancer risk [13]. Multiple studies have also identified associations between CAG repeat length and the risk of ovarian [14], breast [15,16] and endometrial cancers [17,18]. In these studies, the allele patterns were defined as the length of the CAG repeat region. For instance, Terry *et al.* (2005) found that women with of two long *AR* alleles (≥22 CAG repeats) had an increased risk of ovarian cancer compared with those possessing two short *AR* alleles (<22 CAG repeats) [19]. However, the results varied with differences in population and sample size [17,18].

Deletions on the X chromosome often occur in female cancers. Deletions at Xq25 have been reported to be associated with ovarian cancer [20,21], and a high frequency of loss of heterozygosity (LOH) has been found on chromosome arm Xq. The study of LOH with a series of polymorphic microsatellite markers at Xq25 in breast cancer patients found that LOH of DXS8098 was associated with larger tumour size (>3 cm), higher histological grade, and axillary lymph node metastasis. This suggests that there could be one or more TSGs in this region and that the DXS8098 locus on Xq25 could be used as a prognostic marker for breast cancer.

According to Knudson’s two hit hypothesis, we hypothesized that female breast cancer patients may have a high incidence of LOH at Xq25 and that their tumour tissues would exhibit non-random XCI. The occurrence of breast cancer may result from inactivation of a TSG in this region. When a mutated X chromosome with LOH at Xq25 is active while the normal X chromosome is inactive; cancer may eventuate. Then, we can examine whether there might be a candidate tumour suppressor gene at Xq25.

## Methods

### Patients and samples

Seventy-nine breast cancer patients, 39 female lung cancer patients, 30 female other cancer patients (including gastric and liver cancer patients) from Changhua Christian Hospital and 77 healthy females were analysed. The 77 healthy females were divided into an old healthy control group (32–73 years, average 50.81 ± 11.32 years) and a young healthy control group (20–29 years, average 22.86 ± 2.24 years). Tumour and adjacent normal tissues were obtained after surgery and immediately stored in liquid nitrogen or at −70°C for further DNA extraction. The research was approved by Changhua Christian Hospital Institutional Review Board and informed consent was obtained from all patients.

### Microsatellite analysis

A panel of 11 microsatellite markers spanning Xq25 was used for LOH analysis (DXS8067, DXS1001, DXS8059, DXS8098, DXS8009, DXS1047, DXS8068, DXS8078, DXS8071, DXS8041, and DXS8074). These primer sequences were obtained from the NCBI (http://www.ncbi.nlm.nih.gov/projects/genome/guide/human/.

DNA extracted from tumour tissues or adjacent normal tissues of cancer patients was amplified using these microsatellite markers. PCR reactions were performed in a GeneAmp PCR system 2700 thermal cycler (Applied Biosystems, Foster City, CA, USA).

LOH analysis was performed using an ABI Prism 3100 Genetic Analyser. Each forward primer was fluorescein-labelled, allowing amplified fragments to be quantified using ABI PRlSM 3100 GeneScan 3.1 software (Applied Biosystems), which calculated the sizes and areas of signal peaks. The allele ratio was determined as the peak height of the smaller allele divided by that of the larger, and Q^LOH^ was then determined by dividing the tumour allele ratio by the normal allele ratio as per the formula Q^LOH^ = (T_1_/T_2_) / (N_1_/N_2_), where T_1_ and N_1_ are the smaller allele peak heights in the tumour DNA and in the neighbouring normal DNA, respectively, and T_2_ and N_2_ are the larger allele peak heights in the tumour DNA and in the neighbouring normal DNA, respectively. As previously reported, an arbitrary normal range of Q^LOH^ was set from 0.75 to 1.33with a Q^LOH^ lower than 0.75 or greater than 1.33 was defined as LOH [22].

### XCI pattern analysis

The *AR* gene is located on the X chromosome and was chosen as an XCI marker. DNA (200 ng) was digested with 10 U *Hha*I (Fermentas) at 37°C for 12 hr and then heated at 95°C for 10 min to terminate the reaction. The DNA was cleaned by MultiScreen-PCR (96-Well Filtration System, Millipore) and 20 μL ddH_2_O was added to dissolve the DNA. Control experiments were performed in the same manner except that *Hha*I was not added during the digestion period. The PCR reactions and fragment analysis were carried out as previously described [11]. The XCI pattern (random or non-random) was determined by the modified allelic cleavage ratio (CR) of *AR*, as calculated by dividing the smaller peak area ratio by that of the larger. While the Lyon hypothesis, predicts that a normal CR should be 1.0, in reality the XCI pattern is slightly skewed [23]. It is widely accepted that an arbitrary normal value of CR (random XCI pattern) ranges from 0.33 to 3.00 [24]. Therefore, a CR lower than 0.33 or greater than 3.00 was defined as a non-random XCI pattern.

### Statistical analysis

The χ^2^-test, Fisher’s exact test, and Yate’s correction for continuity were used for statistical analysis of the results. Two-tailed *p* values of <0.05 were considered statistically significant.

## Results

### Frequency of LOH in breast cancers

**Figure 1:**
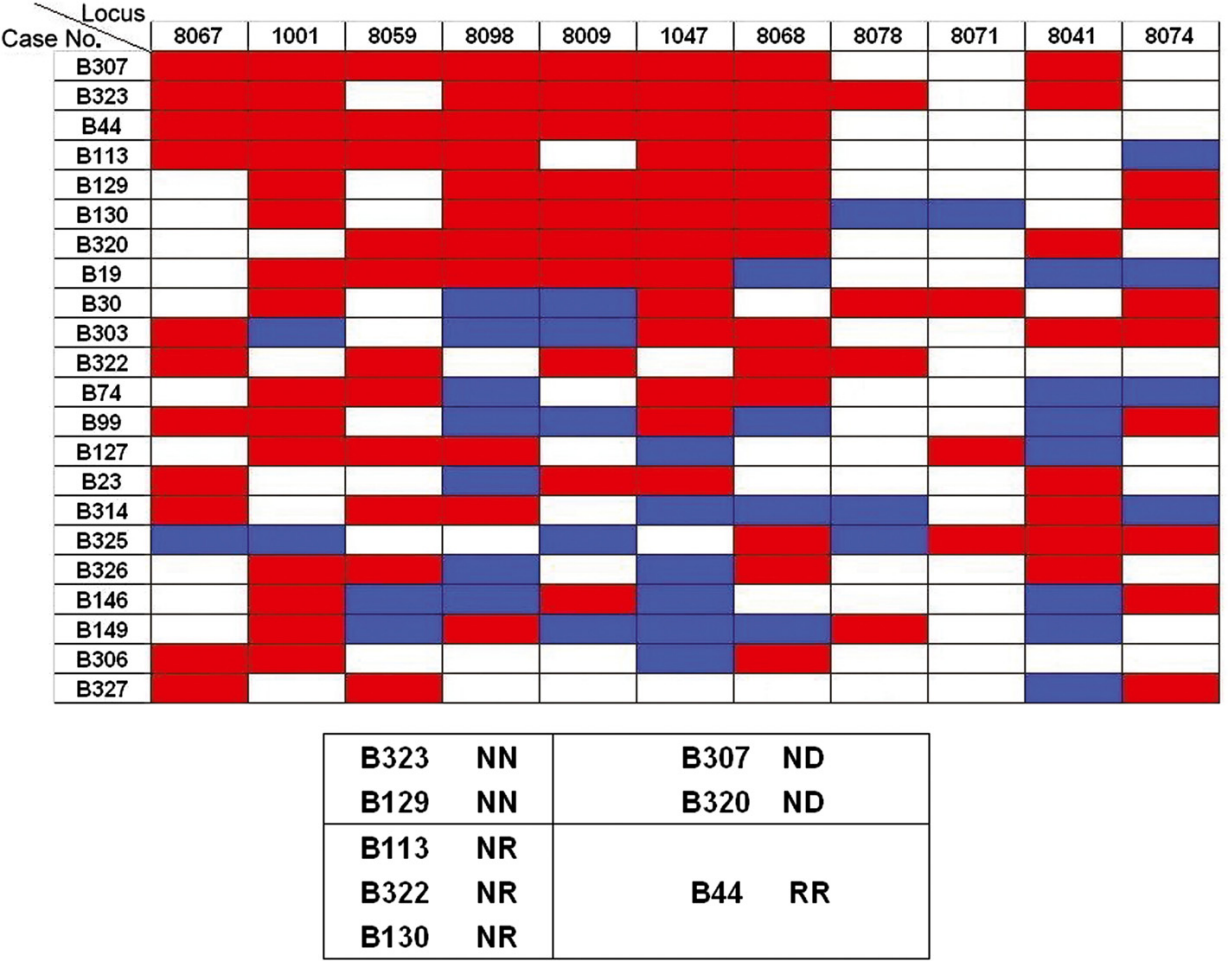
**An explanatory chart of LOH.** Eight breast cancer tissues exhibited LOH at all of the informative loci tested at Xq25, and their XCI patterns tended to be non-random. The figure demonstrates the results of LOH by microsatellite analysis at Xq25. The horizontal axis indicates the position of the 11 microsatellite makers and the vertical axis indicates the case numbers of the 22 breast cancers. Legend: Red rectangles, LOH was detected; open rectangles, retention of heterozygosity; blue rectangles, uninformative cases.

The panel of 11 microsatellite markers at Xq25 was used to analyse Q^LOH^ in tumour DNA obtained from breast and other cancer patients, and the results are as presented in Table 1. The highest frequency of LOH was observed at DXS1047, with 47.23% (26/55) of the breast cancer tissues showing allelic loss, followed by DXS8067 (44.83%), DXS8068 (39.22%), DXS8059 (38.24%), DXS8074 (34.4%), DXS8071 (30.8%), and DXS1001 (30.36%). The frequency of LOH at other microsatellite markers was below 30%. The critical deletion region was around DXS1047 (47.23%).At least one microsatellite locus at Xq25 showing LOH was detected in the tumour DNA of 46 (70.7%) of the 65 breast cancers. Eight of those 65 samples (B44, B113, B130, B129, B307, B320, B322, and B323) showed LOH at all the informative loci tested at Xq25, whereas 38 others showed partial (interstitial or telomeric) alterations at Xq25. A schematic map of LOH at Xq25 in these breast cancer patients is shown in Figure 1. LOH at Xq25 was not identified using these markers in the remaining 19 cases. These results indicate that a commonly deleted region at Xq25 exists in breast cancer patients and that the highest frequency of LOH occurred at the DXS1047 loci.

**Table 1:** Frequency of heterozygosity and LOH in breast cancer and other cancer tissue

**Sample**	**Breast Cancers (BC)**				**Other Cancers (OC)**		***P***
**marker**	**Heterozygosity/No. of BC**	**Heterozygosity %**	**LOH/informative**^**a**^	**LOH**^**b**^** %**	**LOH/informative**	**LOH %**	**value**^**c**^
DXS8067	29/65	44.6%	13/29	44.83%	5/17	29.4%	0.47
DXS1001	56/65	86.2%	17/56	30.36%	6/19	31.6%	0.85
DXS8059	34/65	52.3%	13/34	38.24%	1/8	12.5%	0.17
DXS8098	46/64	71.9%	13/46	28.24%	7/17	14.1%	0.50
DXS8009	50/64	78.1%	14/50	28.00%	6/16	37.5%	0.68
DXS1047	55/65	84.6%	26/55	47.23%	5/20	25.0%	0.14
DXS8068	49/65	78.5%	20/51	39.22%	5/16	31.2%	0.78
DXS8078	20/59	33.9%	4/20	20.00%	1/14	7.1%	0.379
DXS8071	13/58	22.4%	4/13	30.77%	0/14	0.0%	0.041
DXS8041	46/58	79.3%	9/46	19.57%	4/21	19.0%	0.676
DXS8074	32/58	55.2%	11/32	34.38%	2/15	13.5%	0.175
**Xq25**			**46/65**	**70.7%**	**10/25**	**40.0%**	**0.014**

The frequency of heterozygosity and LOH in the breast cancers (BC) and other cancers (OC) are presented in this table.

a. informative: patients with heterozygosity.

b. LOH %: number of patients with LOH divided by number of patients with heterozygosity.

c. Chi-square test and Fisher’s exact probability test.

### Frequency of LOH in the other cancers

The same 11 microsatellite markers at Xq25 were used to detect LOH in the 25 patients with other cancers, including lung and stomach cancers (Table 1). The highest frequency of LOH was observed at DXS8009, with 37.5% (6/16) of these tumour tissues showing allele loss. The frequencies of LOH at DXS1001, DXS8068 and, DXS8067 were about 30%, while the ratio of LOH at other microsatellite markers was quite low at below 20%. Furthermore, no evidence of LOH at DXS8071 was detected in these tissues. At least one Xq25 microsatellite locus showed LOH in 40.0% (10/25) of these other cancer tissues, and in 70.7% (46/65) of the breast cancer tissues (*p* = 0.014).

### The XCI pattern in the tumour tissues and adjacent normal tissues

**Figure 2:**
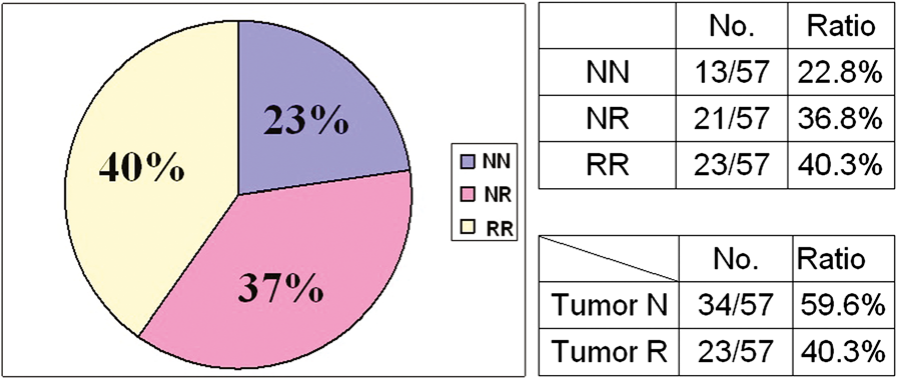
**XCI Pattern in breast cancer tissues.** XCI pattern in the 57 breast cancer patients’ tumour and adjacent normal tissues (RR, NR or NN). Thirteen breast cancer patients’ XCI patterns were NN (22.8%), 21 were NR (36.8%), and the other 23 were RR (40.3%).

**Figure 3:**
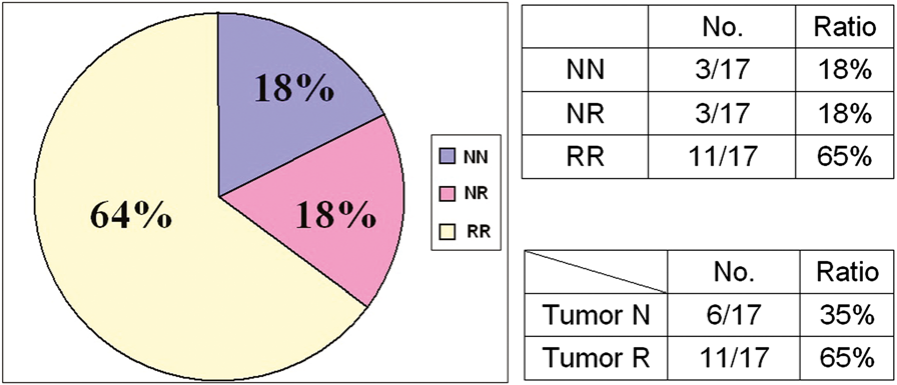
**XCI Pattern in non-breast cancer tissues.** The XCI pattern in the 17 other cancer patients’ tumour and adjacent normal tissues (RR, NR or NN). Three other cancer patients’ XCI patterns were NN (18%), 3 were NR (18%), and 11 were RR (65%).

The XCI pattern was defined as random or non-random, as previously described [25]. Thirteen breast cancer patients’ XCI patterns were NN (non-random in tumour tissue/non-random in adjacent normal tissue; 22.8%), 21 were NR (non-random in tumour tissue/random in adjacent normal tissue; 36.8%), and the other 23 were RR (random in tumour tissue/random in adjacent normal tissue; 40.3%). The XCI patterns in the tumour and adjacent normal tissues of the 17 other non-breast cancer patients were 3 NN (18%), 3 NR (18%), and 11 RR (65%; Figures 2 and 3).

### Relationships between microsatellite analysis, XCI pattern, and clinical parameters

Eight of the 65 breast cancer samples (B44, B113, B130, B129, B307, B320, B322, and B323) showed LOH at all of the informative loci tested at Xq25. The XCI patterns of B129 and B320 were NN, those of B113, B322, and B130 were NR, and B44 was RR. The patterns of XCI in B307 and B323 were not able to be detected because of homozygosity. Interestingly, the patterns of XCI in the tumour tissues of these eight breast cancers tended to be non-random.

An analysis of the relationships between microsatellite analysis, XCI pattern, and clinical symptoms (including tumour grade, lymph node metastases, and tumour size) is presented in Tables 2 and 3. A correlation analysis of LOH pattern and tumour clinicopathological characteristics revealed that LOH at Xq25 or DXS1047 was not associated with higher histologic grade or axillary lymph node metastasis (Table 3).

**Table 2:** Relationships between microsatellite analysis, XCI pattern, and clinical parameters

**Patients in which no LOH was observed at any microsatellite markers**			**Patients in which LOH was observed at one or more microsatellite markers**			
	**No.**	**%**		**No.**	**%**	***P *** **value**^**e**^
**R**^**a**^	11/15	73.3%	**R**^**a**^	20/36	55.56%	0.35
**N**^**b**^	4/15	26.7%	**N**^**b**^	16/36	44.44%	
Tumour Grade 1.2	15/19	78.9%	Tumour Grade 1.2	30/44	68.18%	0.55
Tumour Grade 3	4/19	21.1%	Tumour Grade 3	14/44	31.82%	
LNM^c^+	13/19	68.4%	LNM^c^+	22/45	48.89%	0.25
LNM^d^–	6/19	31.6%	LNM^d^–	23/45	51.11%	
Tumour Size (<3 cm)	8/17	47.1%	Tumour Size (<3 cm)	22/45	48.89%	0.88
Tumour Size (>3 cm)	9/17	52.9%	Tumour Size (>3 cm)	23/45	51.11%	

This table presents the relationships between microsatellite analysis, XCI pattern, and clinical parameters (including tumour grade, lymph node metastases, and tumour size).

a. N: The XCI pattern in the tumour tissues was non-random.

b. R: The XCI pattern in the tumour tissues was random.

c. LNM+: Patients with lymph node metastases.

d. LNM–: Patients without lymph node metastases.

e. Chi-square test and Fisher’s exact probability test.

**Table 3:** Relationships between microsatellite analysis (DXS1047), XCI pattern, and clinical parameters

**Patients in whom no LOH was found at the DXS1047 microsatellite marker**			**Patients in whom LOH was found at the DXS1047 microsatellite marker**			
	**No.**	**%**		**No.**	**%**	***P *** **value**^**e**^
**R**^**a**^	14/24	58.3%	**R**^**a**^	10/20	50%	0.80
**N**^**b**^	10/24	41.7%	**N**^**b**^	10/20	50%	
Tumour Grade 1.2	22/28	78.6%	Tumour Grade 1.2	15/25	60%	0.24
Tumour Grade 3	6/28	21.4%	Tumour Grade 3	10/25	40%	
LNM^c^+	17/28	60.7%	LNM^c^+	12/28	42.9%	0.28
LNM^d^–	11/28	39.2%	LNM^d^–	14/28	57.1%	
Tumour Size (<3 cm)	11/28	39.3%	Tumour Size (<3 cm)	14/26	53.8%	0.28
Tumour Size (>3 cm)	17/28	60.7%	Tumour Size (>3 cm)	12/26	46.2%	

This table presents the relationships between microsatellite analysis (DXS1047), XCI pattern, and clinical parameters (including tumour grade, lymph node metastases, and tumour size).

a. N: The XCI pattern in the tumour tissues was non-random.

b. R: The XCI pattern in the tumour tissues was random.

c. LNM+: Patients with lymph node metastases.

d. LNM–: Patients without lymph node metastases.

e. Chi-square test and Fisher’s exact probability test.

### AR cytosine, adenine, guanine repeats

We estimated the frequencies of *AR* alleles to evaluate the association between *AR*-CAG repeats and breast cancer risk. The CAG repeats of the breast cancer samples were compared with those of similarly-aged healthy controls, and women with two long *AR* alleles (≥21 CAG repeats) were found to possess an increased risk of breast cancer, while those with two short *AR* alleles (<21 CAG repeats) were likely to be normal (*p* = 0.00069; 95% confidence interval 0.5 ~ −0.13). However, when comparing breast cancer patients with young healthy controls, no significant relationships were identified (Table 4).

**Table 4:** Androgen receptor cytosine, adenine, guanine repeats

	**Breast cancer**	**Old controls**^**a**^	**Young controls**^**b**^
(CAG) n, n ≥ 21	42	37	20
(CAG) n, n < 21	2	20	6

In this table, the women with longer CAG repeats (≥ 21) and those with shorter CAG repeats (< 21) are evaluated separately.

a. Old controls: healthy controls of a similar age to the breast cancer patients.

b. Young controls: younger healthy controls.

## Discussion

LOH at Xq25 was determined by examining the frequency of heterozygosity 11 markers in normal tissue DNA and in patients with breast cancer (Table 1). The frequency of heterozygosity of these markers should be greater than 80%. However, some microsatellite markers had a low heterozygosity frequency (DXS8067, DXS8059, DXS8078, DXS8071, and DXS8074), indicating that the allele distribution varies amongst populations. Highly polymorphic markers have a high frequency of heterozygosity which is necessary for suitable analysis of LOH in tumour DNA. The ultimate goal of this project was to identify a candidate TSG. Chromosome arm Xq25 is one of the most frequent LOH regions observed in breast and ovarian cancers [20]. Here LOH of at least one microsatellite locus at Xq25 was identified in 70.7% (46/65) breast cancer patients, which is higher than the percentages reported in previous studies. In comparison, LOH of at least one locus on 7q31 was detected in 34% of sporadic prostate tumours, and the candidate tumour suppressor gene *TES* was found here [26]. LOH of at least one locus on 9q was observed in 56% of transitional cell carcinomas (TCCs) in bladder cancer [27], and LOH of at least one locus at 17q21 (*BRCA1* locus) was found in 60.53% of breast cancers [28]. Candidate TSGs in these regions were subsequently identified. The high frequency of LOH at Xq25 identified in Taiwanese breast cancer patients here indicates that one or more novel candidate TSGs exists at Xq25.

It is possible that one hit to a TSG localized on the X chromosome could be sufficient to induce carcinogenesis because the other allele is inactivated, and several lines of evidence support the hypothesis of the existence of a tumour suppressor gene on Xq25. The X chromosome is involved in the establishment of in vitro immortality and may therefore be important for the control of cell proliferation [29]. In 2002, it was announced that a candidate TSG on the X chromosome was identified using chromosome transfer experiments [30]. Additionally, deletions at Xq25 have been reported to be associated with ovarian cancer [20], and Piao *et al.* found a high frequency of LOH on chromosome arm Xq in breast cancer cells [31]. Piao *et al.* also identified that LOH of DXS8098 at Xq25 is associated with a larger tumour size (>3 cm), a higher histologic grade, and axillary lymph node metastasis, suggesting that there could be one or more TSGs in this region [21]. The frequency of allele loss at Xq25 varies between cancer types, being more common in breast cancers (70.7%) than in other cancers (40%) in our study. These results demonstrate a significant (*p* = 0.014) correlation between LOH at Xq25 and breast cancer risk. The DXS1047 marker showed the highest LOH (47.23%) of the markers tested, and we hypothesized that the regions adjacent to DXS1047 contain one or more candidate TSGs with important roles in breast carcinogenesis but not in other kind of cancers (Table 1).

The XCI patterns in the 57 breast and other cancer tissues were found to be of NN, NR or RR types (Figures 2 and 3). XCI patterns in the breast cancer tumour tissues tended to be non-random, and differed from the other kinds of cancer examined (*p* = 0.047). These results are similar to those of previous studies, in which non-random XCI patterns have been found to be a predisposing factor for the development of invasive ovarian cancer [24].

If a TSG is localized on the X chromosome, a single step, either a mutation or loss of the active allele from the active X chromosome, is sufficient to induce carcinogenesis because the other allele congenitally inactivated. Large deletions at Xq25 were identified in eight of the breast cancer samples with all of the informative loci tested at Xq25 found to have LOH. Additionally, the patterns of XCI of these eight breast cancer tumour tissues tended to be non-random, indicating that the origin of these breast cancer tissues was monoclonal. Thus, carcinogenesis in these patients could be partially explained by two hits to a TSG at Xq25. Here, the first hit is X chromosome condensation and the second is the large deletion at Xq25, which presumably occurs on the active chromosome. In summary, the fact that frequent LOH at Xq25 of the X chromosome was observed in breast cancer tissues suggests that the X chromosome may indeed harbour one or more TSGs, which might play an important role in breast cancer carcinogenesis.

In this study, an increasing number of CAG repeats was found to be associated with elevated breast cancer risk. Multiple studies have previously indicated associations between the length of the CAG repeat and the risk of ovarian [14], breast [15,16] and endometrial cancers [17,18], whereas some breast cancer studies have reported no association [16,32].

We compared the CAG repeat length of the breast cancer patients with those of similarly-aged healthy controls, and found that two long *AR* alleles (≥21 CAG repeats) was associated with an increased risk of breast cancer, while women with two short *AR* alleles (<21 CAG repeats) were likely to be normal (*p* = 0.00069). However, when comparing the breast cancer patients with the young healthy controls, no significant relationship was observed (Table 4). This suggests that the young healthy control individuals with longer *AR* alleles might be at increased risk of developing breast cancer later in life, though they are currently in good health. These results further suggest that longer *AR* alleles may contribute to carcinogenesis in women.

## Conclusion

This study identifies an extraordinary high frequency of LOH at Xq25 in Taiwanese patients with breast cancer. This strongly implies that there might be one or more TSGs related to the development of breast cancer at Xq25 in this population.

## Competing interests

The authors declare that they have no competing interests.

## Authors’ contributions

HTC, YCW and YCC designed the study and performed research. YCW collected data. HCT and STC analysed data. HTC, YCW and YCC wrote the paper. All authors read and approved the final manuscript.

## Pre-publication history

The pre-publication history for this paper can be accessed here:

http://www.biomedcentral.com/1471-2407/14/144/prepub
